# Housing Prices and the Skills Composition of Neighborhoods

**DOI:** 10.3389/fdata.2021.652153

**Published:** 2021-05-31

**Authors:** Shahad Althobaiti, Saud Alghumayjan, Morgan R. Frank, Esteban Moro, Ahmad Alabdulkareem, Alex Pentland

**Affiliations:** ^1^The Center for Complex Engineering Systems at King Abdulaziz City for Science & Technology (KACST) and Massachusetts Institute of Technology (MIT), Riyadh, Saudi Arabia; ^2^Media Lab, Massachusetts Institute of Technology, Cambridge, MA, United States; ^3^Department of Informatics and Networked Systems, School of Computing and Information, University of Pittsburgh, Pittsburgh, PA, United States; ^4^Digital Economy Lab, Institute for Human-Centered AI, Stanford University, Stanford, CA, United States; ^5^Department of Mathematics and Grupo Interdisciplinar de Sistemas Complejos (GISC), Universidad Carlos III de Madrid, Madrid, Spain

**Keywords:** labor economics, urban labor systems, gentrification, labor skills, commuting networks

## Abstract

In the United States (US), low-income workers are being pushed away from city centers where the cost of living is high. The effects of such changes on labor mobility and housing price have been explored in the literature. However, few studies have focused on the occupations and specific skills that identify the most susceptible workers. For example, it has become increasingly challenging to fill the service sector jobs in the San Francisco (SF) Bay Area because appropriately skilled workers cannot afford the growing cost of living within commuting distance. With this example in mind, how does a neighborhood's skill composition change as a result of higher housing prices? Are there certain skill sets that are being pushed to the geographical periphery of a city despite their essentialness to the city's economy? Our study focuses on the impact of housing prices with a granular view of skills compositions to answer the following question: Has the density of cognitive skill workers been increasing in a gentrified area? We hypothesize that, over time, low-skilled workers are pushed away from downtown or areas where high-skill establishments thrive. Our preliminary results show that high-level cognitive skills are getting closer to the city center indicating adaptation to the increase of median housing prices as opposed to low-level physical skills that got further away. We examined tracts that the literature indicates as gentrified areas and found a pattern in which there is a temporal increase in median housing prices and the number of business establishments coupled with an increase in the percentage of skilled cognitive workers.

## 1. Introduction

Alfred Marshall defined cluster theory in his book Principles of Economics as “concentration of specialized industries in particular localities” where industries tend to cluster to facilitate resources and human capital flows in cities (Marshall, 1890). According to this theory, each city should specialize in the industry best fitted to its natural resources and attract workers with the skills needed for that specialization. One clear example of this theory is the cluster of technology companies and businesses in Silicon Valley, which is now home to programmers, engineers, and investors. This dynamic occurs at the city level as well, where workers cluster around the job opportunities in the cities' central business districts (CBDs). CBDs have been explored extensively in the literature; for example, one study looked at commuting patterns and employment data to explore the CBD of Los Angeles (LA) and found the central downtown CBD as well as three other large sub-centers suggesting a polycentric spatial structure (Giuliano and Small, 1991). In the paper of Manduca (2020), the author finds that the spatial distribution of metropolitan employment in the United States (US) is not centralized nor dispersed but spatially concentrated. Economic transformation has a significant effect on the layout and structure of cities and regions. Changes in housing prices can lead to changes in employment and residential areas of workers. Studies are examining the relationship between employment and housing prices. A work by Osei and Winters (2019) examined the effect of labor demand on housing prices and found that the effect varies across metropolitan statistical areas and time.

Changing housing prices and the resulting shift in the spatial distribution of employment are leading factors in gentrification. It significantly affects neighborhood structures and causes social and economic inequalities in urban areas (Christafore and Leguizamon, 2018). Kennedy et al. (2001) define gentrification as “the process by which higher-income households displace lower-income [households] of a neighborhood, changing the essential character and flavor of that neighborhood.” Their study elaborates on the causes of this phenomenon, including increased median housing prices, increased traffic congestion, increased accessibility to city amenities, and rapid job growth alongside economic policies. For example, San Francisco (SF) has experienced rapid job growth in the Silicon Valley alongside a drastic increase in median housing prices, which has led to either gentrification or susceptibility to gentrification in many neighborhoods.

Several studies have investigated the gentrification effect with respect to housing prices. One study considered housing age as an indicator of gentrification in the US, finding that when housing stock is relatively new, it attracts middle-high-income groups to move and push low-income workers out of their neighborhoods (Brueckner and Rosenthal, 2009). The National Community Reinvestment Coalition analyzed the gentrification of neighborhoods for the years 2013–2017 and identified 954 neighborhoods (census tracts) with an indication of gentrification. Their evaluation method included changes in socio-economic data, such as median household incomes, median house prices, and education levels (Richardson et al., 2020). Another study built a model to examine the dynamics of housing prices and the gentrification of neighborhoods. The two main findings included that when poor neighborhoods are located next to wealthier neighborhoods, they experience an increase in housing prices, and their proximity to wealthier neighborhoods will cause gentrification (Guerrieri et al., 2013).

Abundant literature have explored skills in urban labor systems. For example, SkillScape used network science to represent the relationships between skills to uncover their polarization and determine comparative advantages of skills in U.S. cities (Alabdulkareem et al., 2018). Another study that related skilled workers to gentrification tested the hypothesis that full-time skilled workers are likely to live in the city center. They related housing prices to the presence of skilled workers in city centers and referred to skills based on workers' education levels (Edlund et al., 2015). A recently published paper (van Ham et al., 2020) examined the relationship between occupational structure and neighborhood socio-economic segregation to determine whether changes in the occupational structure affect polarization and segregation in cities. The study examined New York, London, and Tokyo, showing that middle-high-income occupations were clustered in city centers, and low-income occupations were often pushed away from city centers.

To investigate the relationship between skills and housing prices, our analysis will focus on a few cities where changes in both variables have occurred recently. According to Storper et al. (2015), LA has had one of the most successful economies. However, income in the SF Bay Area now exceeds the income in LA by 50%. The reasons for this increase have been investigated in Storper et al. (2015). One of them is that the Bay Area experienced economic development that created a demand for new skills. For example, old engineering communities are now occupied by young technologists and academic researchers. In contrast, LA leaders were working in separate environments. Another reason is that leaders in LA advocated for policies to revitalize the old economy, which bolstered the region's increasing low-wage jobs. Investigating these two cities in our analysis gave interesting results that will be shown in the upcoming sections. In addition to these two contrasting cities, we wanted to choose a city where the housing prices have increased significantly. We saw that Dallas, Texas (TX), is a good example. According to the American Community Survey, most of the residents of the Dallas and SF Bay Area commute to work using cars (90 and 77%, respectively). For that reason, we focus on analyzing the neighborhoods of Dallas and the SF Bay Area to have similar commuting patterns across different income groups and workers.

Few studies have looked into the effect of increasing housing prices on workers' skills. Our contribution, in this paper, focuses on the impact of housing prices *via* a granular view of skill compositions to answer the following questions: Has the density of physical skills declined in a gentrified and a high housing-cost area How have recent changes (e.g., gentrification/housing prices) in neighborhoods happened with changes in the skill/job spatial composition in those areas? We hypothesize that, over time, low-skilled workers are pushed away from city centers or areas where highly skilled establishments thrive.

To answer the research questions, we augmented traditional census information (e.g., labor commuting data) with additional detailed information about the skill sets in urban labor markets. While traditional approaches rely only on coarse demographic variables to explain economic growth, we utilized behavioral features (e.g., mobility networks) and detailed worker abilities (e.g., specific skills) to study the effect of gentrification on neighborhoods' skill compositions. Our approach included integrating skills and job networks with various data sources at the census tract level over 17 years (2002–2018) for Dallas, LA, and SF. We used median housing prices and the number of business establishments as metrics of gentrification. We tested for changes in the average travel time and skill compositions of workers at neighborhood levels. Our method included testing spatial autocorrelations for skills and performance regressions to assess the relationship between median housing prices and changes in business establishments and skill compositions. All of the data used in our study are publicly available. Since cities, such as SF, LA, and Dallas have gone through major changes over the last two decades, we focus our study on analyzing them.

## 2. Materials and Methods

### 2.1. Data Overview

Our study utilizes various data sources to examine gentrification from different perspectives (skills, housing prices, and mobility) at the neighborhood level. A summary of the data used can be found in Table 1.

**Table 1:** Data overview summary.

**Data source**	**Data**	**Time resolution**	**Spatial resolution**
LEHD LODES	Commuting patterns.	2002–2018	Tracts
	https://lehd.ces.census.gov/data		
O^*^NET	Skill and occupations.	2018	N/A
	https://www.onetonline.org		
Zillow	Median housing prices.	2002–2018	Zip codes
	https://www.zillow.com/research/data/		
County Business Patterns	No. of business establishments.	2002–2018	Zip codes
	https://www.census.gov/programs-surveys/cbp/data/datasets.html		

#### 2.1.1. Longitudinal Employer-Household Dynamics Origin-Destination Employment Statistics

The Center for Economic Studies at the U.S. Census Bureau offers various data products and resources for public and research purposes, including Longitudinal Employer-Household Dynamics (LEHD) (Graham et al., 2014; Bureau, 2021b). The data offered include information on Job-to-Job Flows, Origin-Destination Employment Statistics, Post-Secondary Employment Outcomes, and Quarterly Workforce Indicators. The Center also offers geographical and temporal coverage for most U.S. states. The LEHD Origin-Destination Employment Statistics (LODES) dataset is based on administrative records and surveys. It includes rich information about employees' home and work location relationships, including how many jobs move from home census blocks to workplace census blocks, with a division of earnings and age groups. Earnings data are categorized into low (< $1,250), medium ($1,250–$3,333), and high (>$3,333) groups. The data provide more information on the demographic and business profiles for residential and workplace census blocks. For residential areas, they provide data on race, ethnicity, education level, and the number of jobs for each of the 20 North American Industry Classification System (NAICS) sectors. For workplace areas, they provide data on the size and age of firms in the 20 NAICS sectors, covering most states for the years 2002–2018.

#### 2.1.2. Occupational Information Network

The Occupational Information Network (O^*^NET) is an online database that provides a detailed description of the U.S. labor market. It is created by job analysts and surveys to identify the skills, tasks, and technologies associated with an occupation in the US. It also provides information on the share of jobs associated with the 20 NAICS sectors. The database is updated regularly (O^*^NET, 2021).

#### 2.1.3. Housing Prices (Zillow)

Zillow (2021) produces a monthly median housing price called Home Value Index (ZHVI), a smoothed, seasonally adjusted measure of the typical home value and market changes over different regions and type of housing. It reflects the typical value for homes in the 35th to 65th percentile range. Their dataset is updated regularly.

#### 2.1.4. County Business Patterns

County Business Patterns (CBP) (Bureau, 2021a) is an annual series that provides subnational economic data by industry. This series includes the number of business establishments. Statistics are available on business establishments at different levels, including zip code level. The series includes almost all NAICS industries. It also excludes the business establishments that reporting government employees. CBP basic data items are extracted from the Business Register (BR), a database of all known business establishment employer companies maintained and updated by the U.S. Census Bureau. The BR provides the most complete and up-to-date data for business establishments. A variety of automated and analytical edits are applied on CBP to remove anomalies and validate the industry's geographical coding and classification.

### 2.2. Methods

#### 2.2.1. Data Collection and Curation

Our study analyzed each city at the geographical resolution of a census tract, which is equivalent to a neighborhood, and used annual temporal resolution (2002–2018). We explored the core-based statistical areas (CBSA) of Dallas-Fort Worth-Arlington, the SF Bay Area, and LA. The CBSA of Dallas has 1,312 tracts, with an average commuting flow of 539,758 pairs between tracts over 17 years, and SF has 1,620 tracts, with an average commuting flow of 759,139 workers. As for LA, we used CBSA boundaries with 3,925 tracts and an average commuting flow of 2,280,330. When addressing these various data sources, we needed to curate and integrate different datasets to aid our analysis. Therefore, we categorized our data curation and mining approach into three categories: skills, housing prices, and business establishments.

##### 2.2.1.1. Skill Processing

Our objective in skills processing is to obtain a skill distribution for each tract. LODES residential area characteristics (RAC) files provided the employment distribution of the 20 NAICS sectors, and O^*^NET provided the percentage of employment for each occupation for the 20 NAICS sectors. With data from the SkillScape analysis (Alabdulkareem et al., 2018), we designated a skill group for each occupation, whether socio-cognitive or sensory-physical, for 672 occupations in the U.S. labor market. We then calculated the ratio of socio-cognitive or sensory-physical skills for each NAICS sector. These ratios were multiplied by the RAC distribution of the 20 NAICS sectors to calculate the number of workers for each skill group and then obtain the cognitive skill fraction.

##### 2.2.1.2. Housing Prices and Business Establishments Processing

In these two categories, we want to get the median housing prices and the number of business establishments for each tract. We used the Zillow dataset for median housing prices and CBP datasets for business establishments. These two datasets are only available for the zip code level, so to convert it to tract level, we used the Department of Housing and Urban Development - United States Postal Service (HUD-USPS) ZIP Code Crosswalk data. Using the crosswalk data, we were able to generate the median housing prices and the number of business establishments for each tract. Also, we used the Consumer Price Index (Statistics, 2021) to adjust the median housing prices for inflation.

#### 2.2.2. Data Analysis

##### 2.2.2.1. Spatial Autocorrelation of the Cognitive Fraction

Exploring how skill compositions have changed over time in a neighborhood requires testing for the first law of geography: “everything is related to everything” (i.e., when there is a concentration of a variable in one area, it is highly likely their neighbors are affected and would follow a similar concentration). In our study, we tested for changes in the spatial autocorrelation of the cognitive fraction over time within tracts. We calculated both the Global Moran's index to obtain the overall trend of a city and the local Moran's index to see the patterns of hot spots (tracts with high cognitive fraction values surrounded by high cognitive fraction value tracts) and cold spots (tracts with low cognitive fraction values surrounded by low cognitive fraction value tracts) of the cognitive fraction within the cities. We used queen contiguity-based weights to define the spatial neighbors and the cognitive fraction as the variable to test its autocorrelation with these spatial weights.

##### 2.2.2.2. Network Analysis

To capture the dynamic behavior of mobility flows, we used techniques from network science to understand how different income groups travel to work overtime. We constructed directed networks for the flows of low- and middle-high-income groups, where each source node represents a home tract and each target node represents a workplace tract. Nodes are connected by the number of workers commuting from home to work based on their income. Calculations of the temporal change of the network through weighted degree measures and centrality help assess flow networks. The weighted degree will provide information on where each income group is constructed in terms of the workplace and how they are shifting over time. We used the weighted eigenvector centrality of all nodes to understand the importance of a tract in commuting the various income groups.

#### 2.2.3. Regression Analysis

In order to examine the relationship between the different variables and the median housing prices over the years, we built a regression model that takes as input cognitive skill fraction, centrality, number of business establishments and produces median housing prices for each census tract.

## 3. Results

### 3.1. Flow Networks

**Figure 1:**
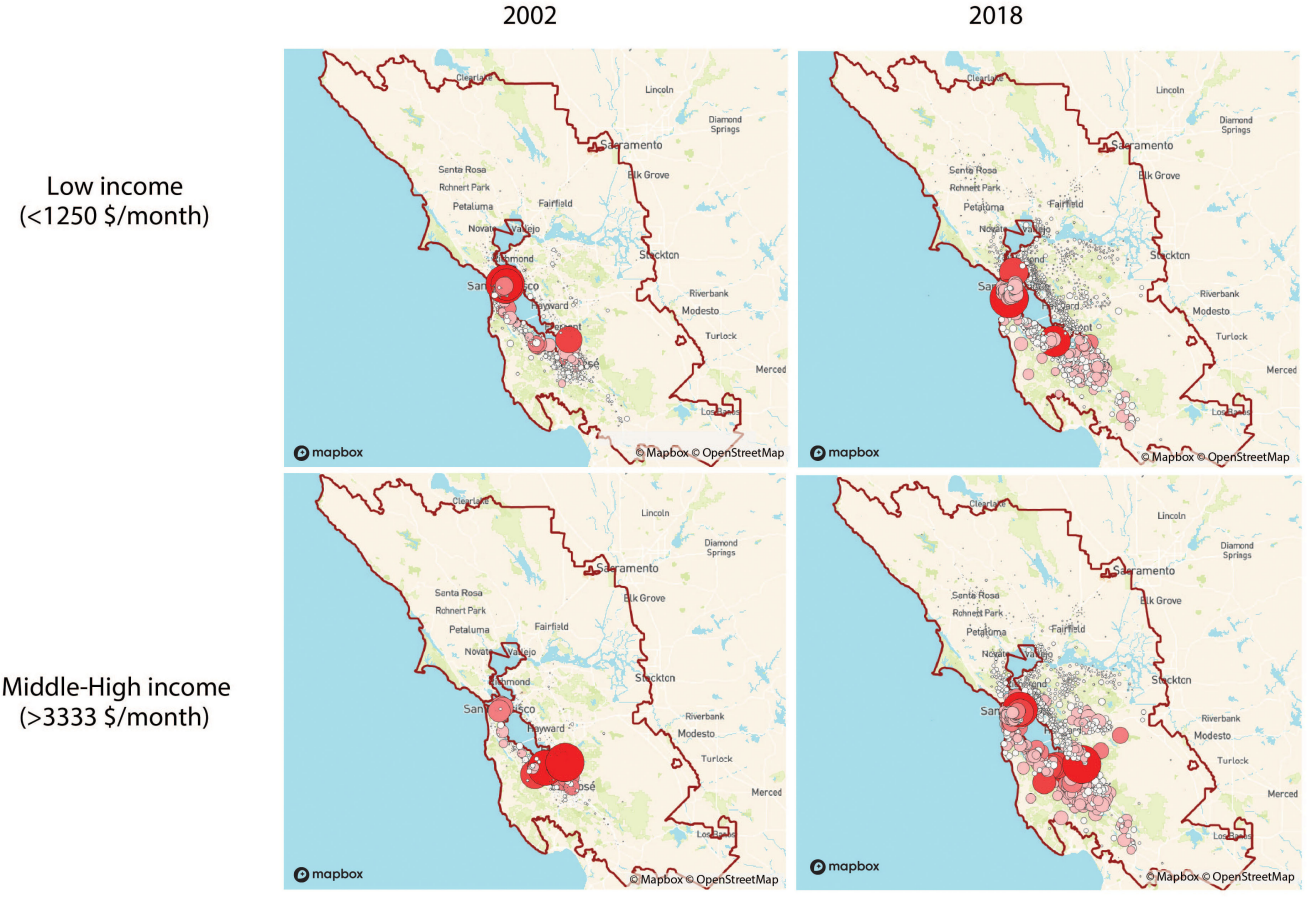
Flow networks of home places of workers commuting to workplaces located in the city centers (around the red circle) based on income groups (weighted out-degree). Node size and color [light (low)-dark (high)] indicates the degree ranking in San Francisco Bay Area.

**Figure 2:**
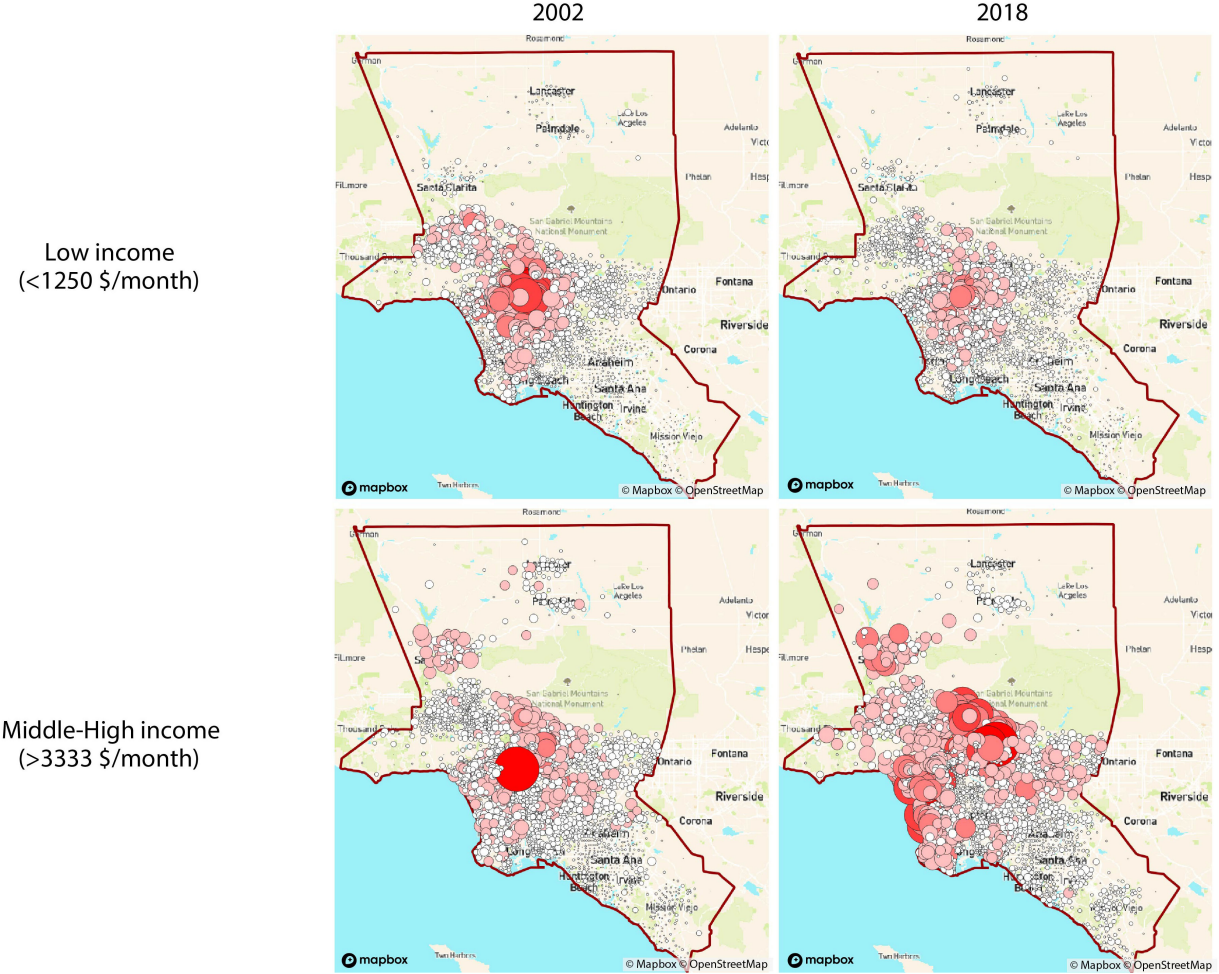
Flow networks of home places of workers commuting to workplaces located in the city centers (around the red circle) based on income groups (weighted out-degree). Node size and color [light (low)-dark (high)] indicates the degree ranking in Los Angeles.

**Figure 3:**
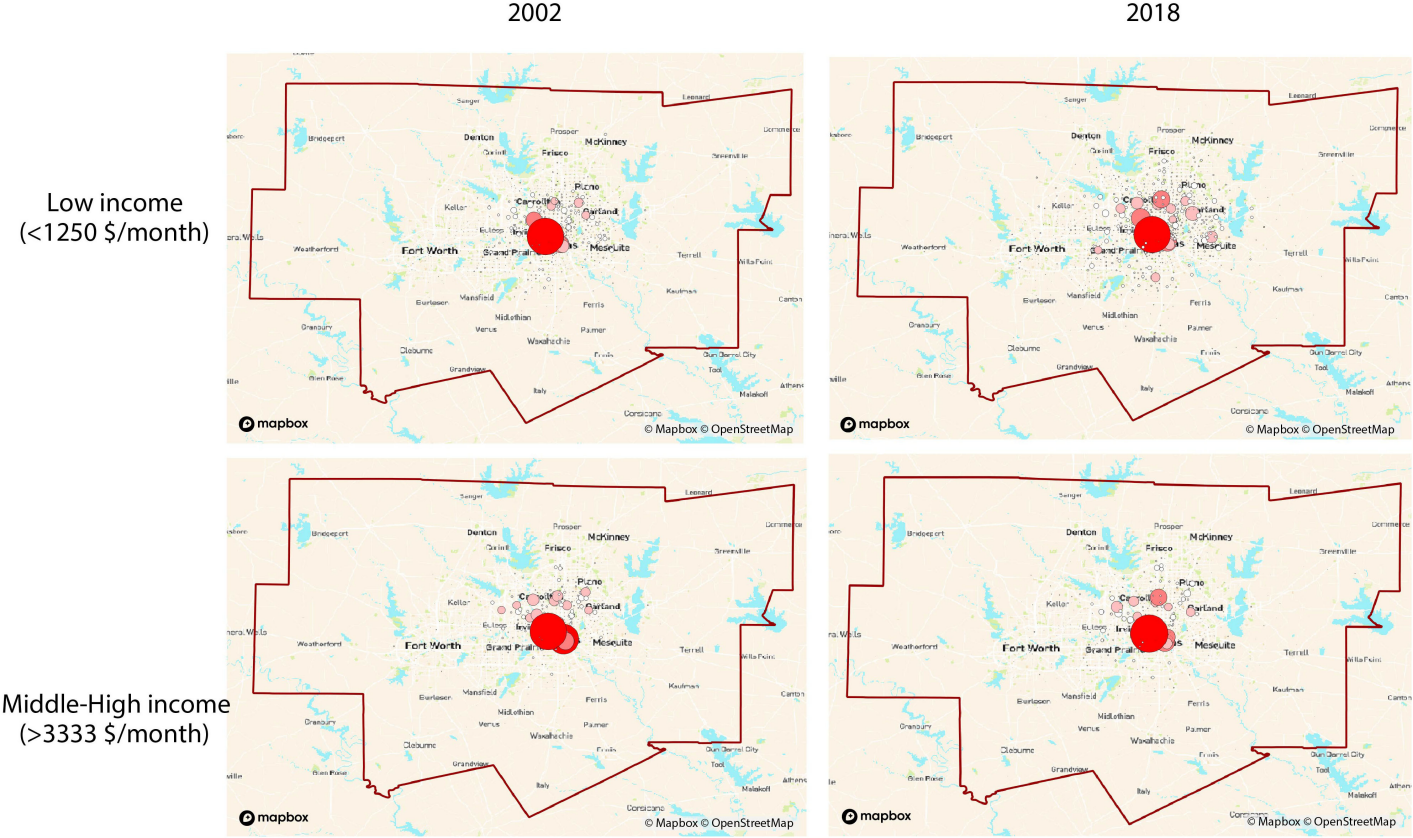
Flow networks of home places of workers commuting to workplaces located in the city centers (around the red circle) based on income groups (weighted out-degree). Node size and color [light (low)-dark (high)] indicates the degree ranking in Dallas.

Network visualizations show the dynamics of how different income groups commute to workplaces that we defined as the city's downtown and Silicon Valley for the Bay Area for the years of 2002 and 2018. We use weighted out-degree networks to capture the changes in the home place of high- and low-income workers. SF Bay area clearly shows the difference in flows between the two groups in Figure 1. SF Bay area clearly shows the difference in flows between the two groups. The low-income group is clustered in the city center of SF during both years. However, it has drastically declined in the area near the Silicon Valley in 2018 and looks more spread than in 2002. On the other hand, the middle-high-income group maintains its cluster around the Silicon Valley during 2002 and 2018. It looks more spatially spread in 2018 than in 2002, and more density has increased in the SF downtown. As for LA commuting patterns, there is a clear difference among the two groups' home areas in Figure 2. The low-income group seems to be concentrated downtown in 2002 and then distributed in 2018. The middle-high-income group had a similar concentration as the low-income group, but in 2018, the home areas show to be located on the coast side and the northern LA area. In Dallas, whose commuting flow is shown in Figure 3, there seems to be no notable difference of home areas of workers commuting to work in Dallas' downtown among groups and years.

### 3.2. Spatial Autocorrelation

For our first question of how spatial composition changed in neighborhoods over the period of 17 years, we assess this by looking into the global spatial autocorrelation (Moran's I) of the cognitive fraction. In particular, to examine if the cognitive skill workers tend to live near each other or not on a temporal level. The results shown in Supplementary Figure 2 indicate a general trend of increasing correlation overtime as it started in Dallas with a correlation of 55% in 2002 to 81% in 2018 and for the Bay area with almost 78–84% for the years 2002 and 2018, respectively and LA has global spatial correlation of 78% in 2002 to 88% in 2018. This indicates patterns of clustering of cognitive workers in home areas. To further understand how these are being clustered in the neighborhoods and examine the areas with statically significant correlations, we used the local indicator of spatial association (LISA) (Anselin, 1995).

**Figure 4:**
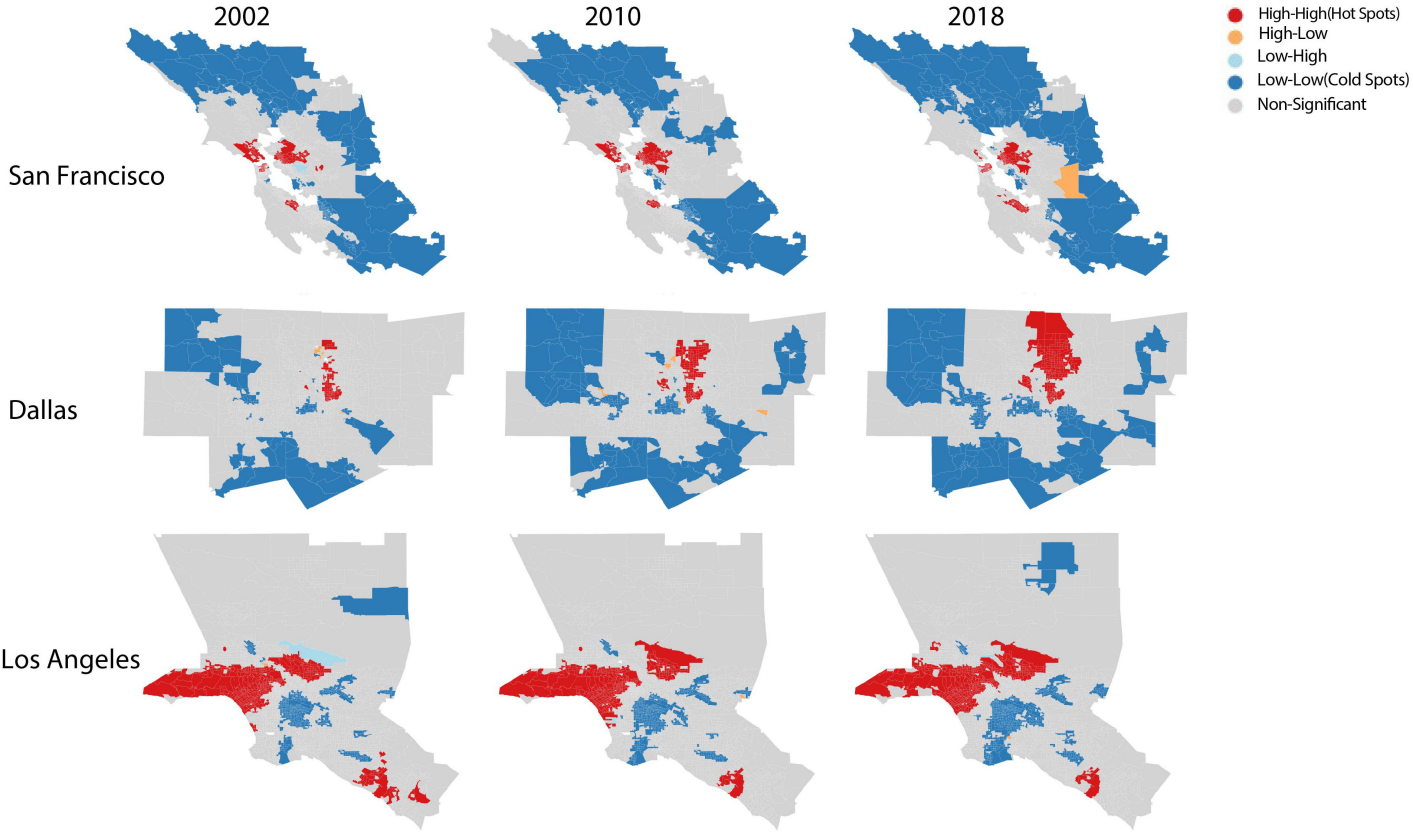
Local spatial autocorrelation significant areas map.

Local indicator of spatial association will identify the significant locations of hot spots and cold spots. Specifically, high values of positive autocorrelation are clustered, and the low values of negative autocorrelation are clustered. The significance LISA maps of the SF Bay Area in Figure 4 show that the area around Silicon Valley has been clustering with the high values of the cognitive fraction. Generally, the hot spots are clustered closer to the SF downtown and the Silicon Valley. As for Dallas, it is clear to see in Figure 4 that over time the areas of hot spots are expanding closer to the center while cold spots are moving further. These patterns show how the skill compositions have changed significantly in the areas closer to the city center. In LA, LISA map shows that tracts with high values of the cognitive fraction are clustering around the wealthiest neighborhoods of LA, such as Beverly Hills and Bel Air. Furthermore, it seems that the significant areas are attracting nearby neighborhoods where they over time became statistically significant in the cognitive fraction.

### 3.3. Regression Analysis

As we find out that cognitive skills cluster in highly gentrified areas, we wanted to test if cognitive skills contribute to housing prices. So, we compared it with another variable that could contribute to the housing prices by comparing different regression models in Table 2. In model 1, we only considered centrality. Model 2 demonstrates the superior performance of cognition (R^2^ = 0.177). Also, we consider the number of business establishments in model 3 (R^2^ = 0.035). Models 4–6 demonstrate that cognitive skill fraction contributes the most between the first three variables. In model 7, we consider variables for the cities. Modeling with these geographical variables alone performs worse than using cognitive (R^2^ = 0.16). Model 8 shows that the time variable almost does not contribute to the dependent variable (R^2^ = 0.002). Model 9 demonstrates the improved performance from including the variable for centrality and establishments (R^2^ = 0.203), but maximum performance is achieved when including cognition as well (model 10 has R^2^ = 0.299). The standard errors and statistical significance of coefficient estimates are reported in the regression table. In summary, we find that the cognitive skill fraction (cognitive) explains the housing prices better than models using centrality, business establishment, city, or time alone.

**Table 2:** Depending on cognitive skills, ordinary least squares (OLS) regression estimates the housing prices.

	**Dependent variable: housing prices**									
	**Model 1**	**Model 2**	**Model 3**	**Model 4**	**Model 5**	**Model 6**	**Model 7**	**Model 8**	**Model 9**	**Model 10**
Cognitive		0.635^***^		0.619^***^	0.602^***^	0.596^***^				0.519^***^
		(0.004)		(0.004)	(0.004)	(0.004)				(0.004)
Centrality	0.524^***^			0.413^***^		0.378^***^			0.488^***^	0.410^***^
	(0.010)			(0.009)		(0.009)			(0.009)	(0.009)
Establishments			0.103^***^		0.046^***^	0.034^***^			0.061^***^	0.019^***^
			(0.002)		(0.002)	(0.002)			(0.002)	(0.002)
City I							−0.049^***^		−0.044^***^	−0.047^***^
							(0.001)		(0.001)	(0.001)
City II							0.049^***^		0.052^***^	0.030^***^
							(0.001)		(0.001)	(0.001)
Year								1.493^***^	1.312^***^	−0.715^***^
								(0.103)	(0.092)	(0.088)
Constant	0.076^***^	−0.267^***^	0.060^***^	−0.263^***^	−0.259^***^	−0.258^***^	0.085^***^	−1.405^***^	−1.243^***^	0.505^***^
	(0.000)	(0.002)	(0.000)	(0.002)	(0.002)	(0.002)	(0.000)	(0.103)	(0.092)	(0.088)
*R*^2^	0.026	0.177	0.035	0.193	0.183	0.196	0.160	0.002	0.203	0.299
Adjusted *R*^2^	0.026	0.177	0.035	0.193	0.183	0.196	0.160	0.002	0.203	0.299

Note: ^*^p < 0.1; ^**^p < 0.01;

*** *p < 0.001. Centrality variable resulted from subsection Network analysis. Cognitive skill fraction (Cognitive) variable, from Skill processing. Business Establishments (Establishments) variable, from Housing prices and business establishments processing. City variables account for the fixed effect of the city; where City I represents Dallas, City II represents San Francisco (SF), and Los Angeles (LA) represented by the constant. All variables were standardized before regression. SEs are reported in parentheses, and asterisks indicate the statistical significance of coefficient approximations. We performed the regression on 99,118 observations and reporting the root mean square error of the resulting model*.

## 4. Discussion and Conclusions

The main novelty of the paper is adding the lens of skills to explore the economic transformation affecting cities and neighborhoods. Other studies have used other proxies to skills, such as income and education. In our work, we used exact skills to quantify the skills changes for each neighborhood. In addition, this study quantifies the spatial impact of skills on commute patterns of middle-high- and low-income groups. We found that low-skill physical workers are being pushed out of the city center, which could eventually hurt the local economy. The work is a building block toward a granular view of specific skills changes at the neighborhood level and further examining their relation to gentrification. This was possible by combining and integrating various data from mobility networks to skills data. The LISA significance maps show how high values of cognitive skill workers have significantly clustered around the city center over time, pushing low values further for both SF and Dallas. However, this trend was not present in LA as it seems to be clustered in wealthy neighborhoods.

Moreover, the global spatial correlation index for the distribution of cognitive fraction has increased between 2002 and 2018 for all areas of study, showing that an apparent change has forced this increase in global correlation. In addition, the flow networks show the difference in commuting patterns between the middle-high- and low-income groups. Furthermore, the regression experiment showed that the cognitive fraction contributed significantly to the median housing prices. This analysis can be done for all U.S. states. The change in cognitive fraction could identify areas to be gentrified and find opportunity zones for investment and future technology hubs to flourish the local economy. To compare between the three cities, we found that LA had the most remarkable average percentage change in cognitive fraction with an average percent increase of 6%, followed by Bay area with a percent increase of 5%, and Dallas with the lowest average percentage change of 3%. However, Dallas had the most significant spatial correlation with a 47% percentage increase in global Moran's I. In contrast, the other regions had a percentage change of 8 and 13% for Bay Area and LA, respectively.

**Figure 5:**
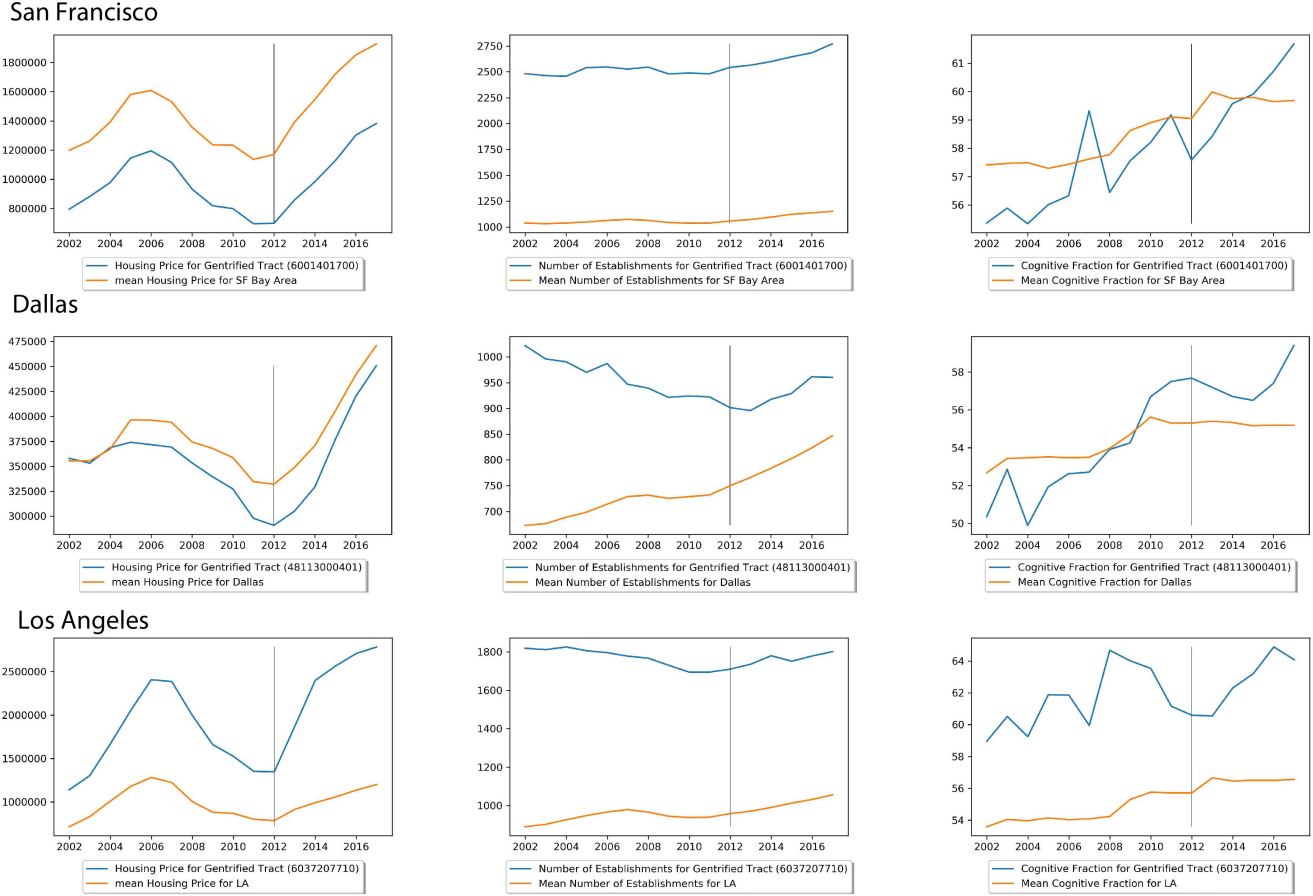
Temporal change in variables of tracts defined by National Community Reinvestment Coalition (NCRC) as gentrified tracts in 2012.

Our work is aligned with the perspective article titled “Toward understanding the impact of artificial intelligence on labor” (Frank et al., 2019) that discusses how skills and employment shape our urban economies. The strength of this study is that it provides a new preservative to examine the effect of gentrification with the lens of skills. However, this study does not look into the cause and effect of housing prices and business establishments, indicators for gentrification, effect skills compositions, or the other way around. Figure 5 displays the changes in median housing prices, skill fractions, and the number of business establishments over time in tracts that NCRC defined as gentrified area compared to the mean of these variables in the area of study. In these examples, it is clear to see the increasing trend in all three variables after these are gentrified. However, examples in Supplementary Figure 6 show that although median housing prices and business establishments have increased, the cognitive fraction has decreased. Causality analysis is needed to examine further the results, and more variables, such as road congestion and travel times would help uncover the reasons for such change.

## Data Availability Statement

The original contributions presented in the study are included in the article/Supplementary Material, further inquiries can be directed to the corresponding author/s.

## Author Contributions

All authors listed have made a substantial, direct and intellectual contribution to the work, and approved it for publication.

## Conflict of Interest

The authors declare that the research was conducted in the absence of any commercial or financial relationships that could be construed as a potential conflict of interest.
