# Supplementary material for: Housing Prices and the Skills Composition of Neighborhoods
Source: Front Big Data. 2021 May 31;4:652153. doi: 10.3389/fdata.2021.652153 (PMC8200666; doi:10.3389/fdata.2021.652153)
Supplement: Supplementary file 1 [file Data_Sheet_1.zip › SupplementaryMaterial/SupplementaryMaterial.pdf]

Supplementary Material

1 SUPPLEMENTARY DATA

The datasets used for this study are all publicly accessed and cited. We also used open source python libraries such as scikit-learn to do the analysis. A repository containing the code is available here: https://github.com/ShahadThobaiti/Housing-Prices-and-the-Skills-Composition. Additional data related to this paper may be requested from the authors.

2 SUPPLEMENTARY TABLES AND FIGURES

2.1 Figures

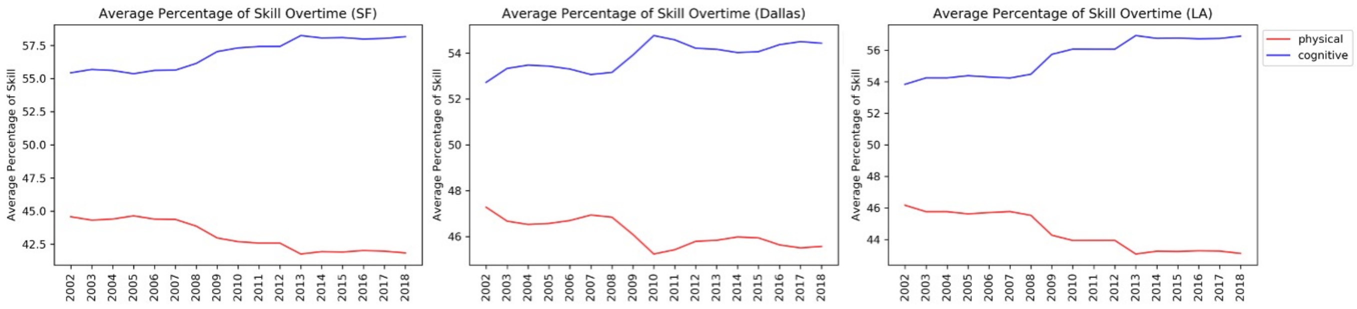

Figure S1: Average cognitive and physical fraction for the areas of study.

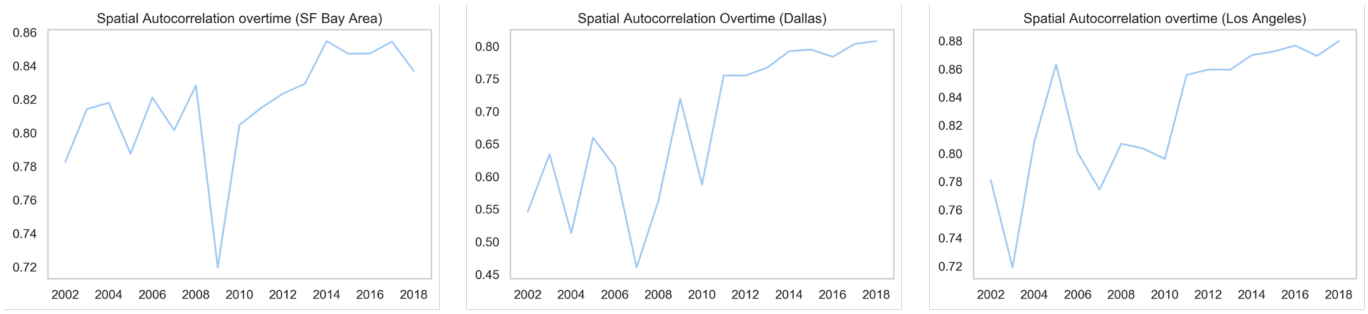

Figure S2: Global spatial autocorrelation index overtime.

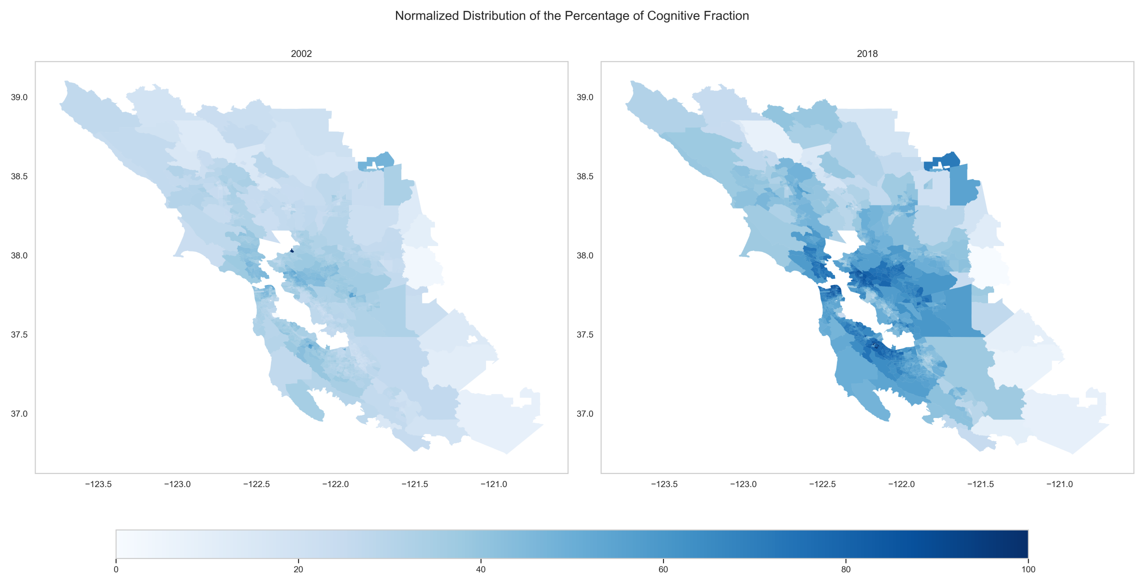

Figure S3: Cognitive skill normalized distribution for San Francisco Bay area low(light) and high(dark).

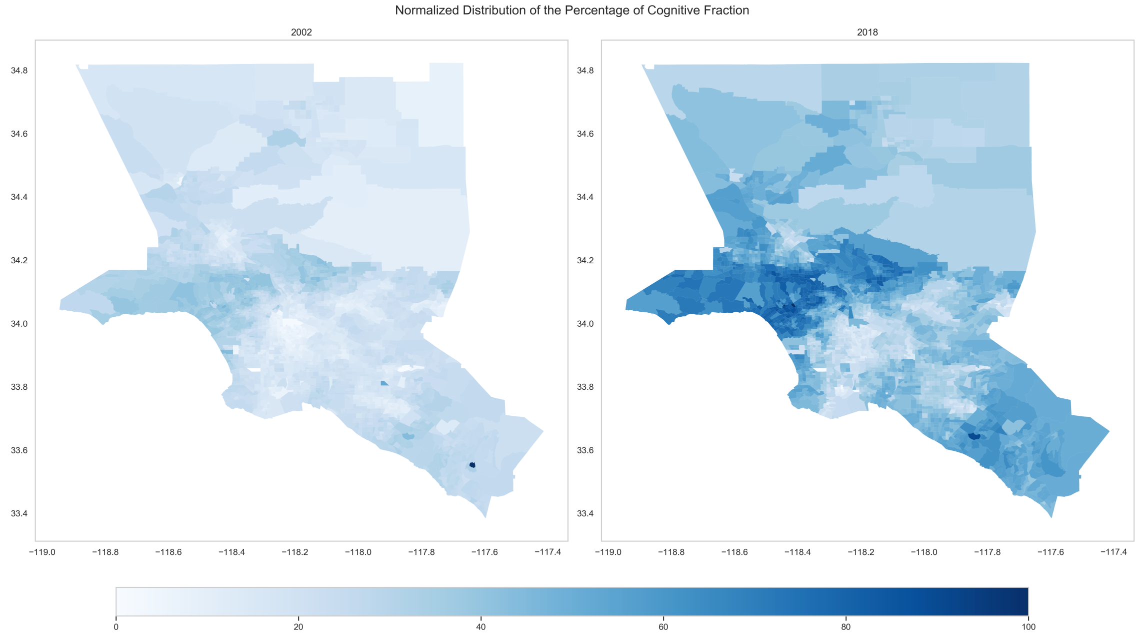

Figure S4: Cognitive skill normalized distribution for Los Angeles low(light) and high(dark).

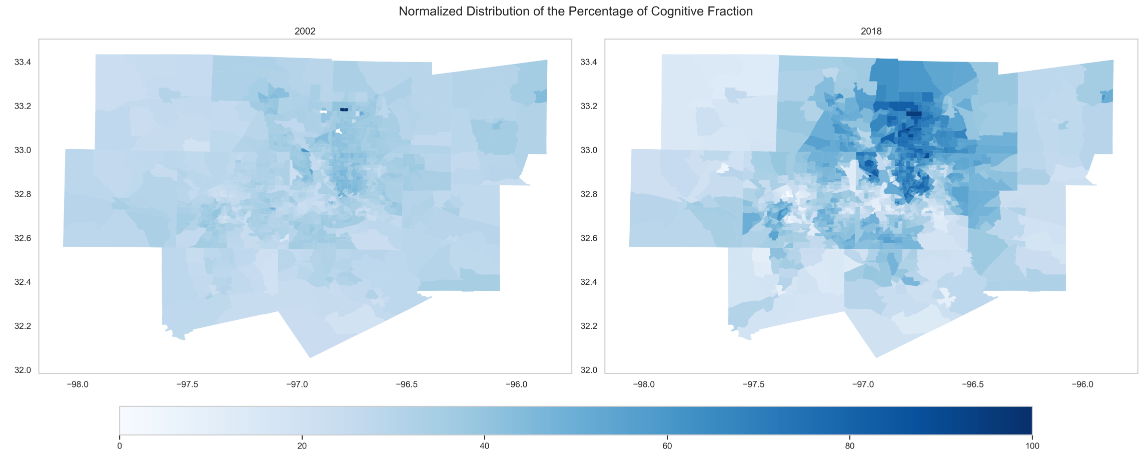

Figure S5: Cognitive skill normalized distribution for Dallas low(light) and high(dark.

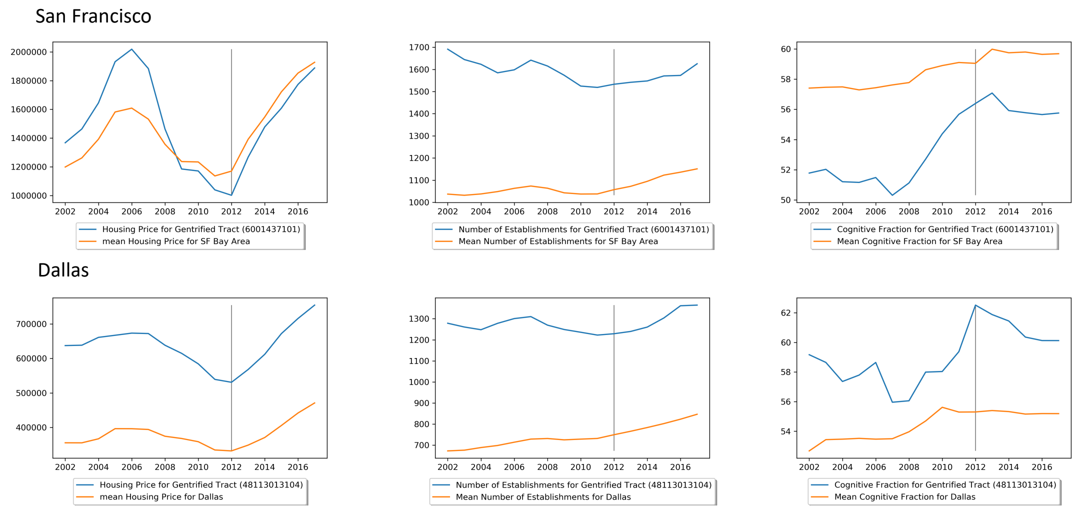

Figure S6: Temporal change in variables of tracts defined by NCRC as gentrified tracts in 2012.
