# Supplementary figures and images for: Housing Prices and the Skills Composition of Neighborhoods
Source: Front Big Data. 2021 May 31;4:652153. doi: 10.3389/fdata.2021.652153 (PMC8200666; doi:10.3389/fdata.2021.652153)

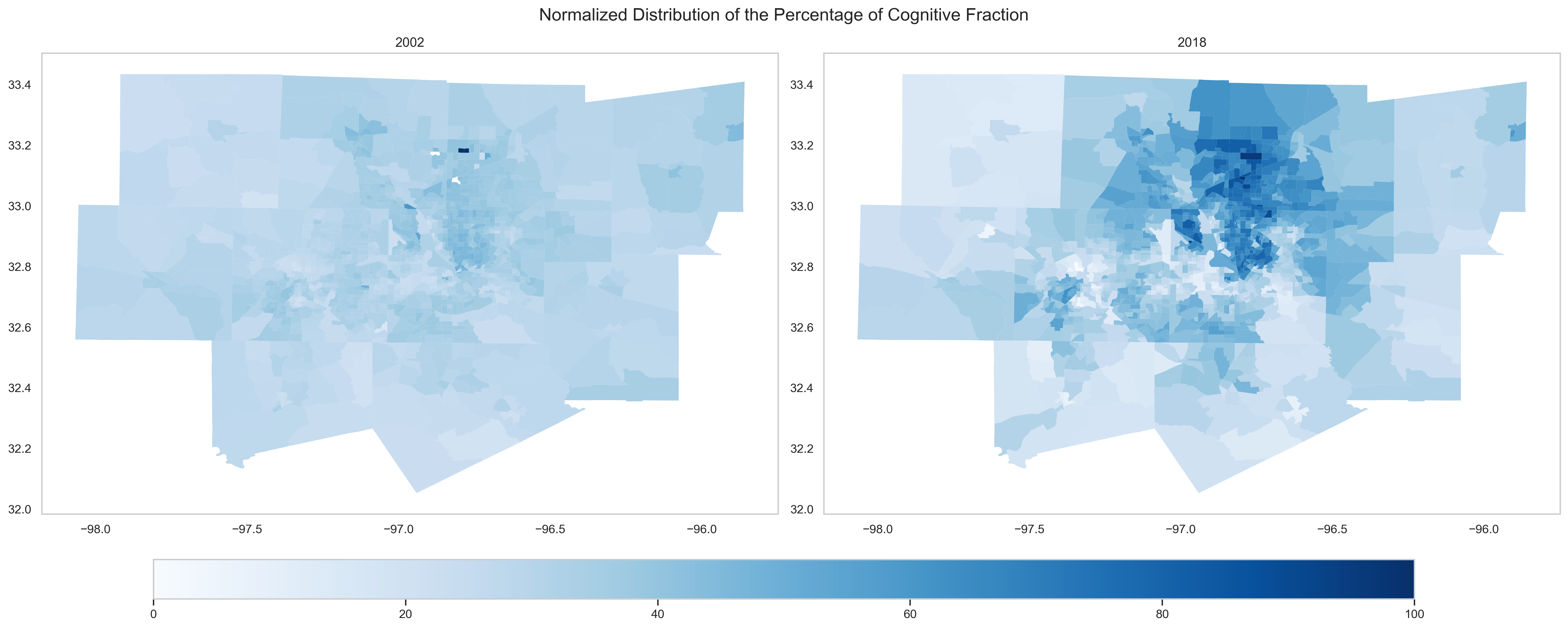

Supplement: Supplementary file 1 [file Data_Sheet_1.zip › SupplementaryMaterial/SI Figures/S5.png]

# San Francisco

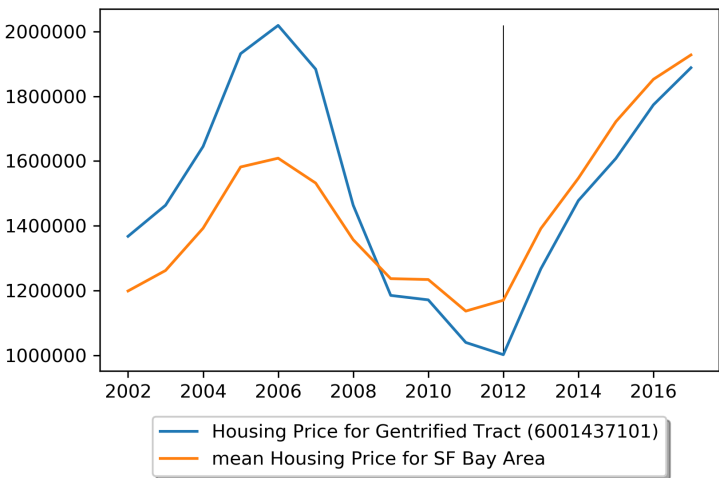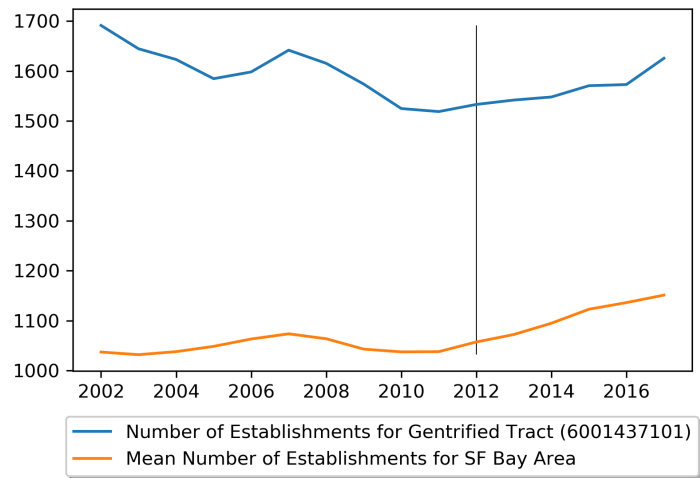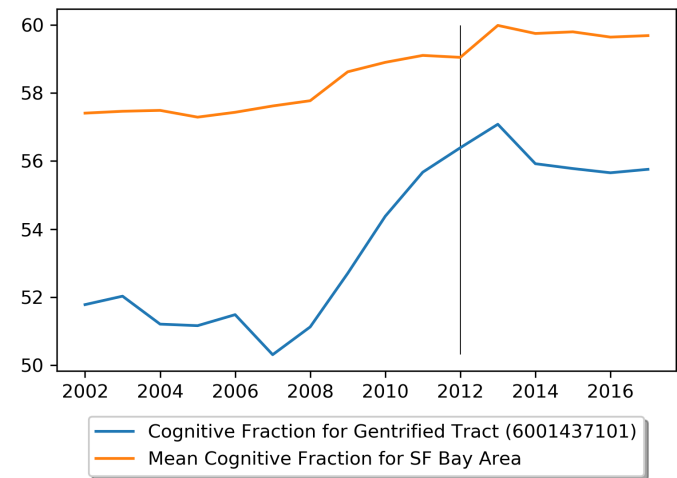

# Dallas

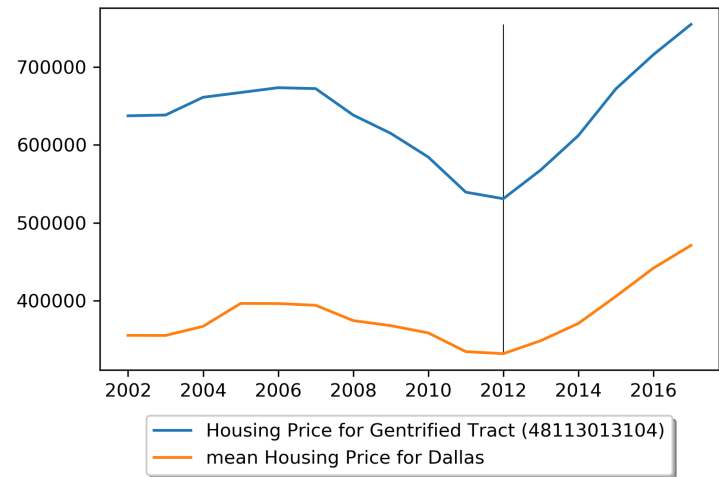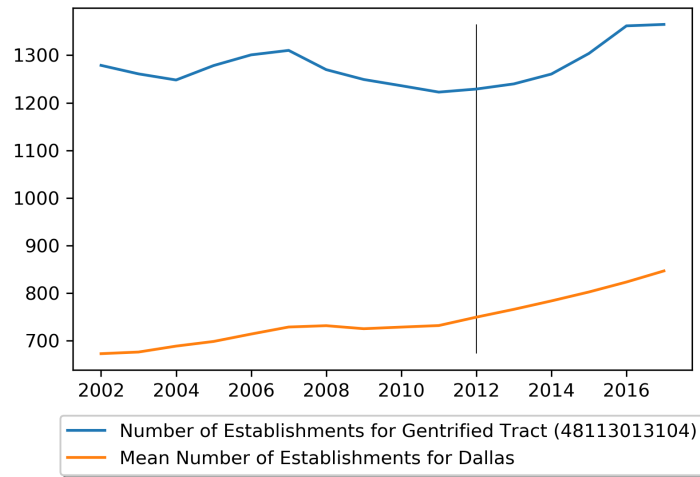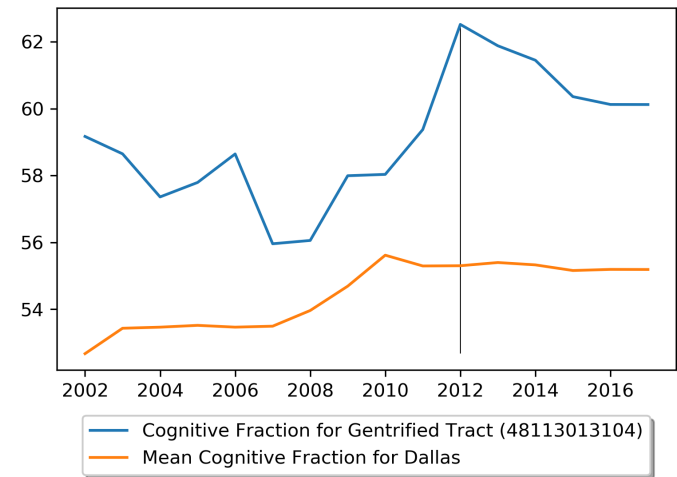

Supplement: Supplementary file 1 [file Data_Sheet_1.zip › SupplementaryMaterial/SI Figures/S6.pdf]

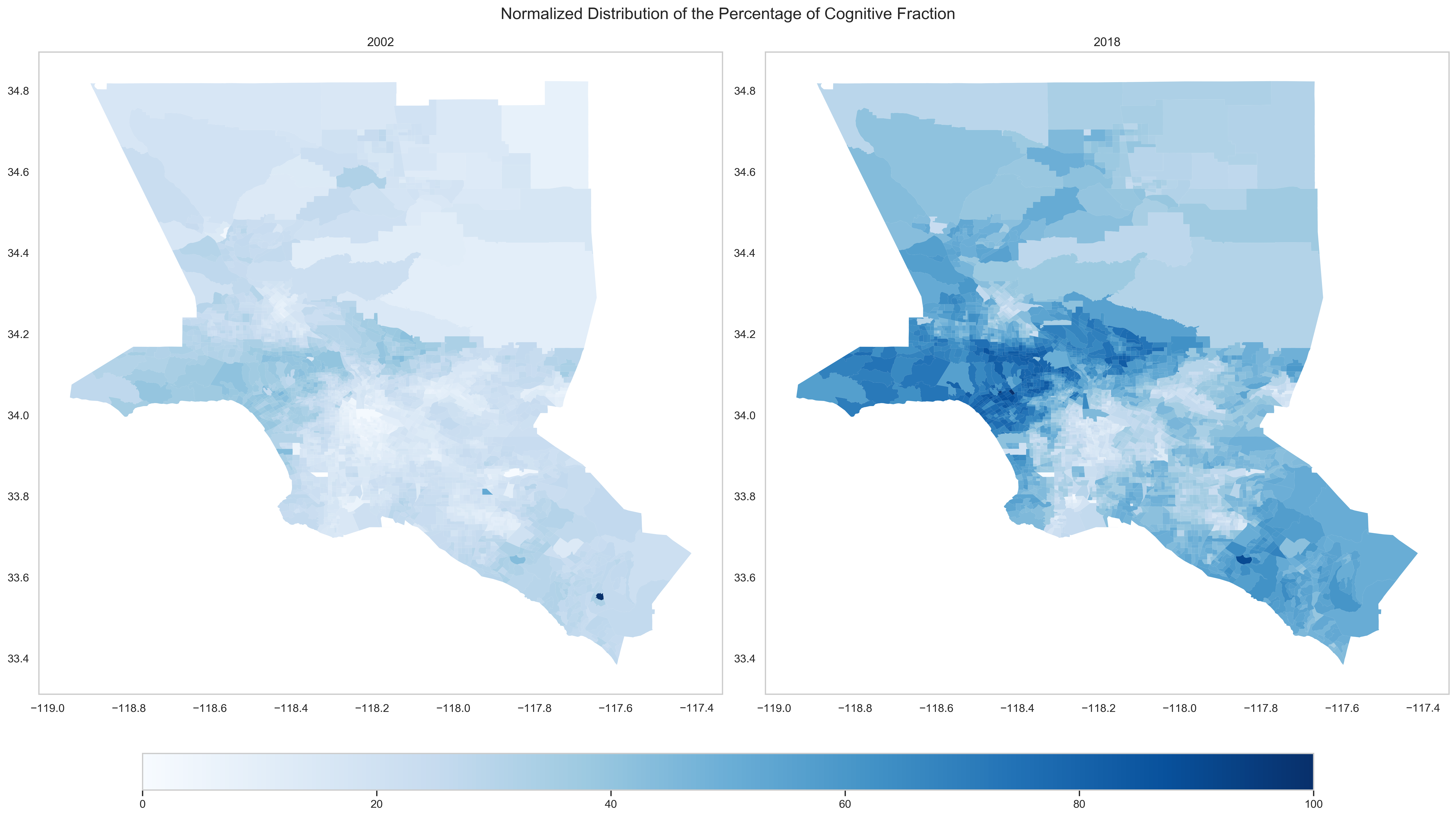

Supplement: Supplementary file 1 [file Data_Sheet_1.zip › SupplementaryMaterial/SI Figures/S4.png]

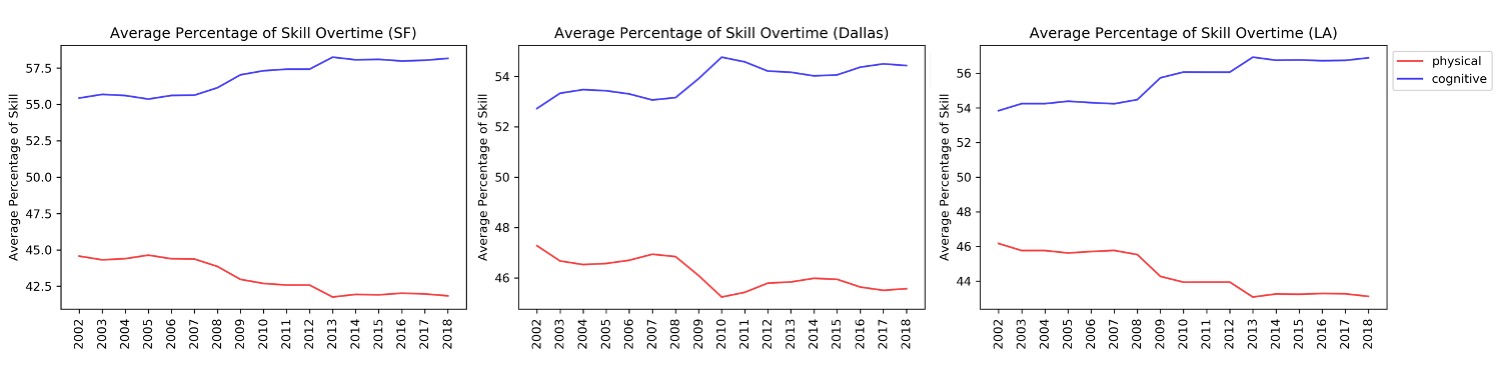

Supplement: Supplementary file 1 [file Data_Sheet_1.zip › SupplementaryMaterial/SI Figures/S1.jpg]

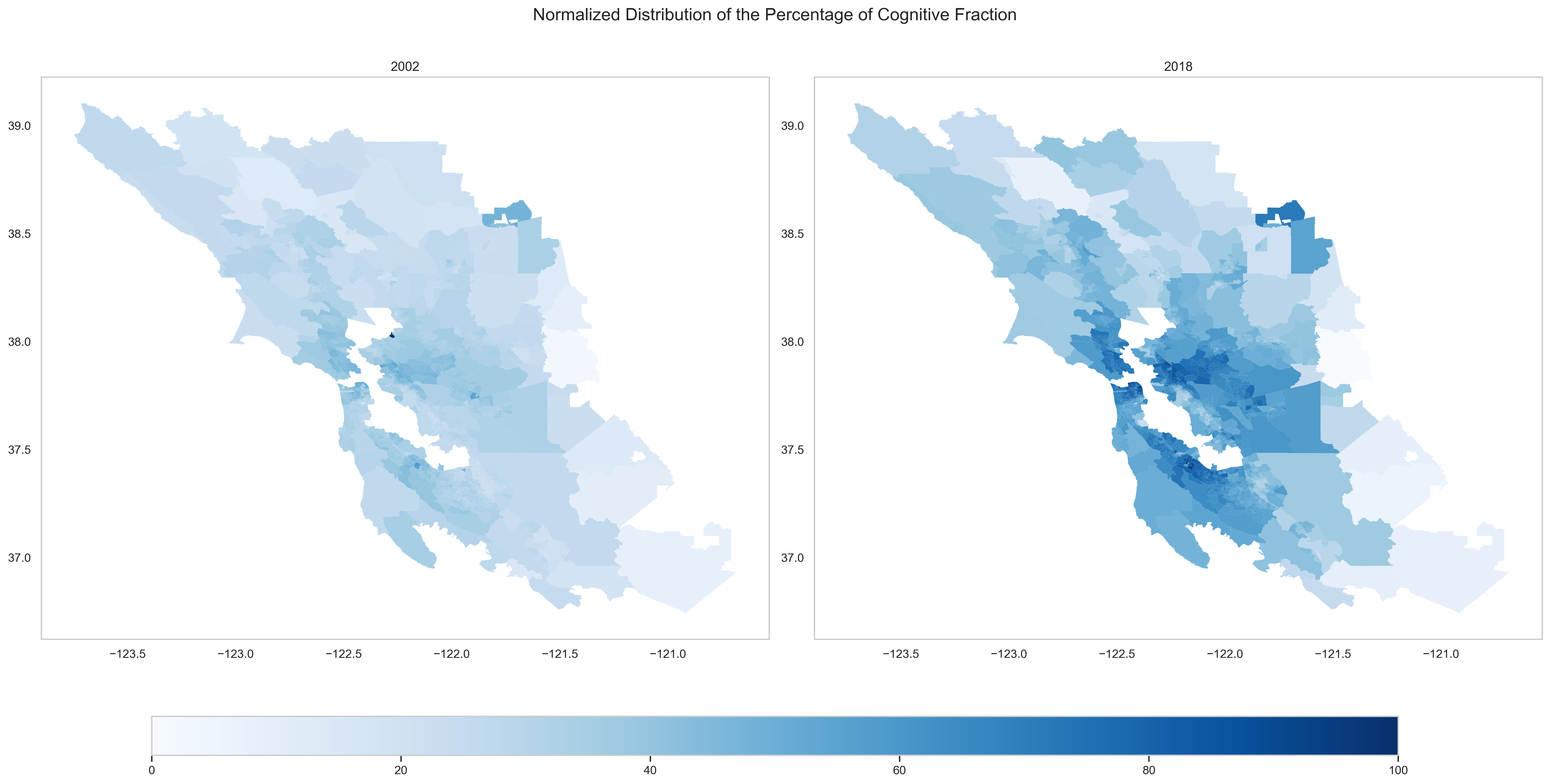

Supplement: Supplementary file 1 [file Data_Sheet_1.zip › SupplementaryMaterial/SI Figures/S3.png]

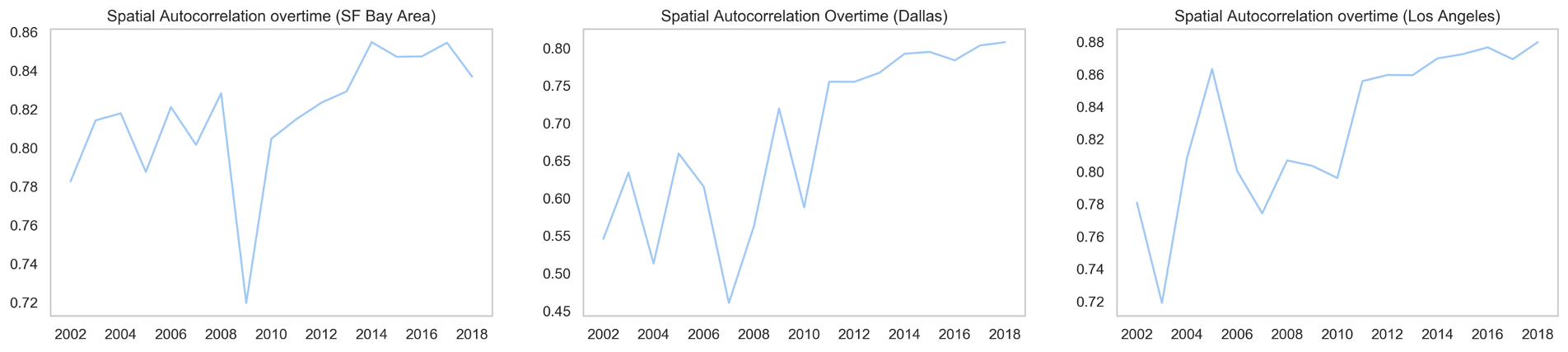

Supplement: Supplementary file 1 [file Data_Sheet_1.zip › SupplementaryMaterial/SI Figures/S2.png]
